# Antibacterial effects of low-temperature plasma generated by atmospheric-pressure plasma jet are mediated by reactive oxygen species

**DOI:** 10.1038/s41598-020-59652-6

**Published:** 2020-02-20

**Authors:** McKayla J. Nicol, Timothy R. Brubaker, Brian J. Honish, Alyssa N. Simmons, Ali Kazemi, Madison A. Geissel, Connor T. Whalen, Christopher A. Siedlecki, Sven G. Bilén, Sean D. Knecht, Girish S. Kirimanjeswara

**Affiliations:** 1 Department of Veterinary and Biomedical Sciences, The Pennsylvania State University, University Park, PA 16802 USA; 2 Pathobiology Graduate Program, The Pennsylvania State University, University Park, PA 16802 USA; 3 School of Electrical Engineering and Computer Science, The Pennsylvania State University, University Park, PA 16802 USA; 4 Department of Mechanical Engineering and Department of Biomedical Engineering, The Pennsylvania State University, University Park, PA 16802 USA; 5 Department of Engineering Science and Mechanics, The Pennsylvania State University, University Park, PA 16802 USA; 6 Department of Surgery, Penn State College of Medicine, Hershey, PA 17033 USA; 7 School of Engineering Design, Technology, and Professional Programs, The Pennsylvania State University, University Park, PA 16802 USA; 8 The Center for Molecular Immunology and Infectious Disease, The Pennsylvania State University, University Park, PA 16802 USA; 9 The Center for Infectious Disease Dynamics, The Pennsylvania State University, University Park, PA 16802 USA

**Keywords:** Microbiology, Medical research, Engineering

## Abstract

Emergence and spread of antibiotic resistance calls for development of non-chemical treatment options for bacterial infections. Plasma medicine applies low-temperature plasma (LTP) physics to address biomedical problems such as wound healing and tumor suppression. LTP has also been used for surface disinfection. However, there is still much to be learned regarding the effectiveness of LTP on bacteria in suspension in liquids, and especially on porous surfaces. We investigated the efficacy of LTP treatments against bacteria using an atmospheric-pressure plasma jet and show that LTP treatments have the ability to inhibit both gram-positive (*S. aureus*) and gram-negative (*E. coli*) bacteria on solid and porous surfaces. Additionally, both direct LTP treatment and plasma-activated media were effective against the bacteria suspended in liquid culture. Our data indicate that reactive oxygen species are the key mediators of the bactericidal effects of LTP and hydrogen peroxide is necessary but not sufficient for antibacterial effects. In addition, our data suggests that bacteria exposed to LTP do not develop resistance to further treatment with LTP. These findings suggest that this novel atmospheric-pressure plasma jet could be used as a potential alternative to antibiotic treatments *in vivo*.

## Introduction

Antibiotics were first introduced to the clinic for use against severe bacterial infections in the early 1940s. By the 1950s, significant antibiotic resistance had been noted^1^. According to the Centers for Disease Control and Prevention (CDC), a minimum of 2 million people are infected each year with antibiotic-resistant bacterial strains in the United States, resulting in nearly 25,000 deaths^2^. The Global Antimicrobial Resistance Surveillance System started by the World Health Organization in 2015 has helped to increase awareness of the severity of the issue of resistance, with incoming data from over 20 countries and reports of over 500,000 isolates demonstrating resistance to different antibiotics^3^. Multiple strategies have been implemented throughout the past several decades to combat the spread of antibiotic-resistant bacteria. Some of these strategies include the introduction of novel antibiotics, alteration of guidelines for recommended frequency and length of antibiotic therapies, improvement of awareness and knowledge base regarding resistant isolates, and incorporation of combination antibiotic regimens^4^. Despite these intervention tactics, the threat of antibiotic-resistant bacterial strains remains relevant on a global scale. Finally, aside from the medical obstacles posed by treatment of patients with bacterial infections, the spread of antibiotic resistance also results in significant excess healthcare system costs and economic burdens with estimates of up to $1 billion per year and $3 trillion in worldwide gross domestic product losses respectively^5,6^.

It is the combination of all of these factors that has necessitated the search for new treatment options targeting bacterial infections. In the past several years, plasma medicine has emerged as a leading potential alternative to antibiotic treatments. Plasma medicine is an interdisciplinary field that combines plasma physics with research in the life sciences to create solutions to a variety of biomedical problems. Recently, there has been an increased focus on the development and implementation of low-temperature plasma (LTP) sources that are able to meet specifications required of medical instrumentation^7^. The well-reported use of plasmas for sterilization of non-biomedical materials has presented an opportunity for plasma medicine to expand into the realm of antiseptics^8–11^. This research has resulted in the successful application of two plasma configurations known as dielectric barrier discharge (DBD) and atmospheric-pressure plasma jets (APPJ)^12^. These configurations have been used for treatment of chronic wounds and multiple dental applications. Additionally, further investigation is being done in several other fields including oncology, ophthalmology, and aesthetic surgery^12^. Another related area within plasma medicine is the use of plasma-activated media (PAM), which has been of particular interest for cancer treatments^13,14^.

Despite the progress that has been made thus far regarding the use of LTP for biomedical applications, there is still much to be explored. The mechanisms of action behind LTP treatments have yet to be fully elucidated. Additionally, unique plasma sources will likely be adapted and optimized based on the targeted medical application. Therefore, there is an acute need for different systems and discharge configurations to be studied further. More importantly, there are only a few studies that have investigated the benefits of either APPJ or DBD configurations in treating drug-resistant bacteria, particularly for *in vivo* settings^9–12,15–19^. Here, we describe the construction of an APPJ system and characterize the bactericidal activity of this system. It was found that LTP treatments were able to consistently produce 90% reduction in both gram-positive and gram-negative bacterial strains on solid and porous surfaces, as well as in liquid. More importantly, our results revealed that generation of reactive oxygen and nitrogen species (ROS/RNS) during LTP treatments was critical for its bactericidal activity, and that bacteria do not develop resistance to LTP treatments. Together, this represents an important step forward towards developing plasma-mediated strategies for combating drug-resistant bacterial infections.

## Results

### Development and operation of atmospheric-pressure low-temperature plasma jet system (APPJ System)

**Figure 1:**
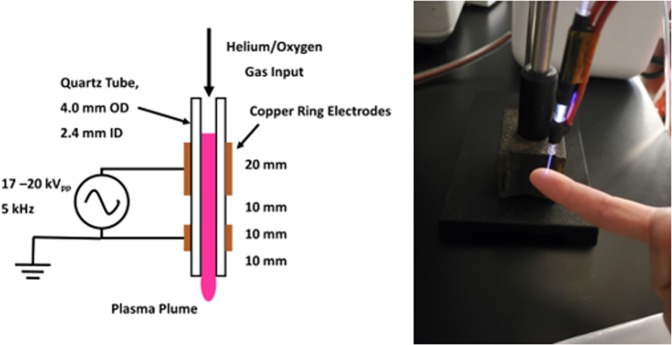
Diagram of APPJ setup. (Left) The APPJ consists of a quartz tube with outer diameter 4 mm and inner diameter 2.4 mm Copper ring electrodes are located on the outside of the tube separated by 10 mm and with the ground electrode located 10 mm from the jet exit. Helium with or without oxygen admixture flows through the tube and is excited by a high-voltage amplifier. (Right) The APPJ can be exposed to living tissue without thermal sensation, demonstrating that its effects are non-thermal in nature.

**Figure 2:**
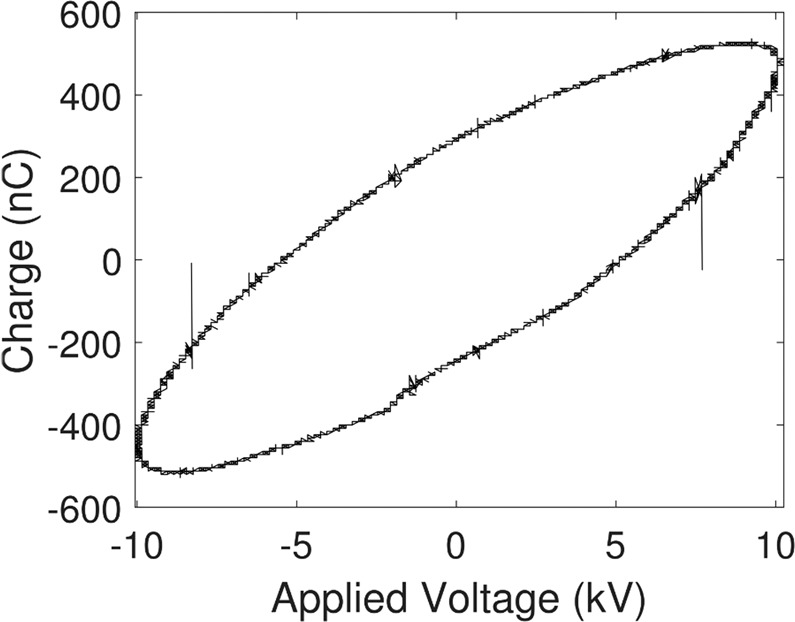
Lissajous plot of APPJ. The standard Lissajous plot shows the charge on the monitor capacitor (*C* = 10 nF) placed between the downstream electrode and the electrical ground of the high-voltage amplifier. Integrating the enclosed area and multiplying by the frequency provides the power deposited in the plasma jet.

We developed a quartz-based plasma jet with copper electrodes, supplied by helium and oxygen gases, with the goal of producing plasma with minimal thermal discharge. While the design and technical details of the jet are discussed in materials and methods section, Fig. 1 (left panel) depicts a schematic of the APPJ system^20^. Figure 1 (right) shows the jet during operation exposed to living tissue, demonstrating the non-thermal nature of the device. The Lissajous method^21^ was used to determine the power consumption of the discharge, which was found to be between 7.5–11 W, depending on the operating voltage. We found that the jet could be operated safely at voltages up to 20 kV_pp_, which is the limit of our amplifier system. A representative Lissajous plot is shown in Fig. 2 for 10 kV_pp_ operating conditions at a frequency of 5 kHz.

### LTP treatments inhibit the growth of gram-positive and gram-negative bacteria

**Figure 3:**
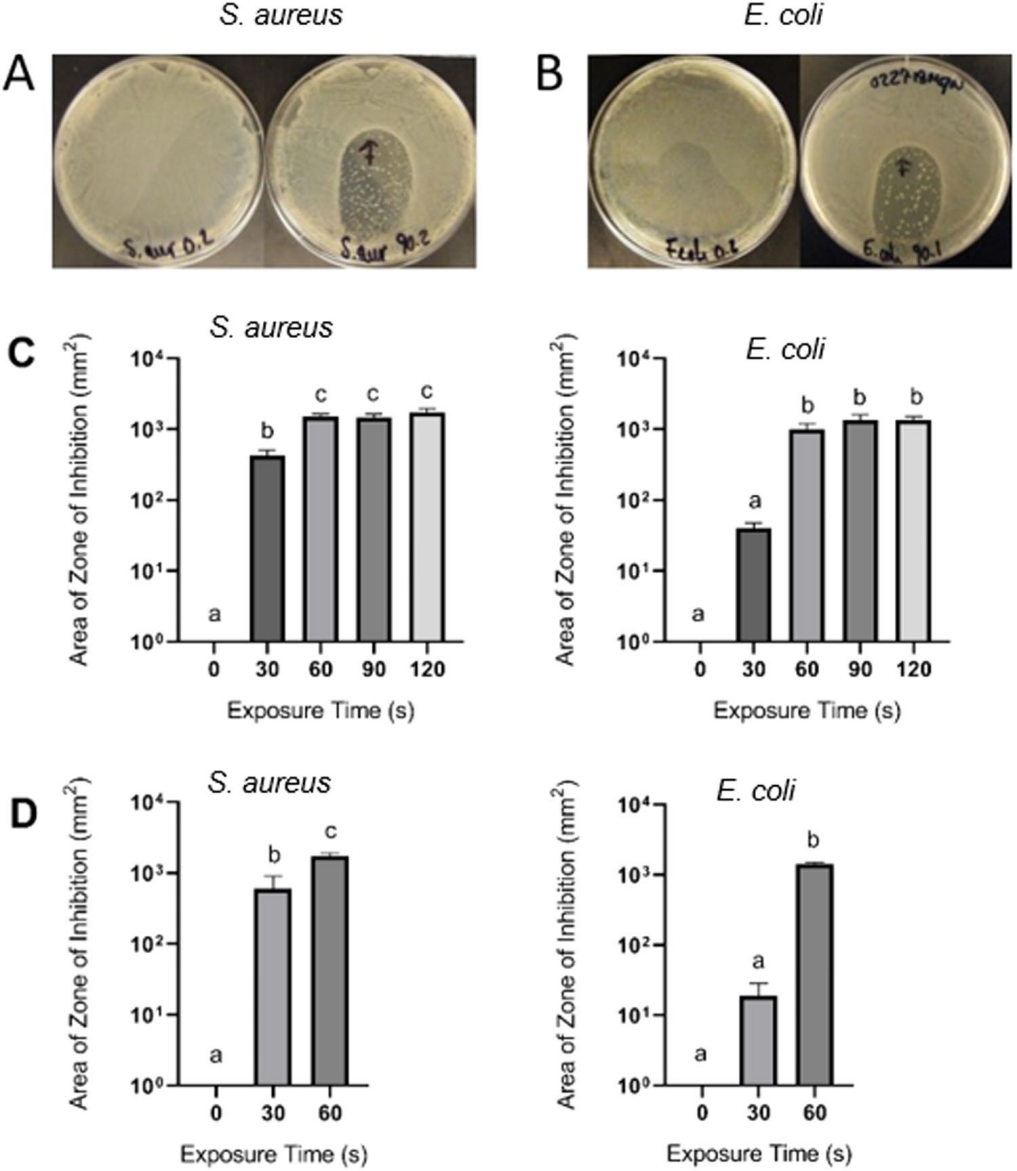
Low temperature atmospheric pressure plasma jet inhibits bacterial growth of *S. aureus* and *E. coli*. (**A**) Representative images of untreated (left) and treated (right) LB plates with 5 × 10^7^ CFU/mL of *S. aureus* and (**B**) *E. coli*. (**C**) Quantification of zones of inhibition of *S. aureus* and *E. coli* from 0, 30, 60, 90, and 120 s of exposure to LTP, N = 5. (**D**) Quantification of area of zones of inhibition of indicated bacteria plated in soft agar and exposed to 0, 30, and 60 s of LTP. Statistical significance was determined by one-way ANOVA with Tukey’s multiple-comparison test. Significance is shown via grouping: a = significantly different from groups marked with b or c; b = significantly different from groups marked with a or c; c = significantly different from groups marked with a or b p ≤ 0.05, N = 3.

LTP treatments have previously been shown to inhibit the growth of bacteria both on hard surfaces and in liquid environments^8–12,14–17,19,22–24^. Therefore, we investigated the effect of our APPJ system on both gram-positive and gram-negative bacteria, *Staphylococcus aureus* and *Escherichia coli*, respectively. When these bacteria were plated on LB agar and exposed to the LTP stream for 90 s, a significant reduction in the growth of both bacteria was observed around the zone of exposure to LTP (Fig. 3, panels A and B). Subsequently, the areas of the zones of inhibition were measured as a function of the duration of LTP exposure to quantify the effect of LTP on bacterial growth (Fig. 3, panel C). It was found that 30 s of exposure was sufficient for a significant reduction in the growth of *S. aureus* (Fig. 3C, left panel). A 60 s exposure to LTP resulted in a statistically significant reduction in the growth of *E. coli* (Fig. 3C, right panel), although a trend towards reduction in growth was noted after 30 s exposure (p ≤ 0.05). Additionally, exposure times longer than 60 s did not further reduce the growth for either bacteria. To investigate if these LTP treatments were effective for non-surface level applications, bacteria suspended and grown in soft agar were subjected to similar LTP treatments. A more porous surface with bacteria suspended throughout was used to more accurately simulate *in vivo* conditions of a deep tissue infection. While small zones of inhibition were observed after 30 s of treatment, a more significant inhibition was noted after 60 s of exposure for both *S. aureus* and *E. coli* (Fig. 3D). Several system parameters such as voltage, distance of jet from biological samples, and ratios of gaseous mixtures (oxygen: helium) were tested to optimize the system for maximum effect on bacteria with no thermal effects. It was found that decreases in voltage (Fig. S1) and flow rate (Fig. S2) as well as increases in distance between the jet nozzle and sample (Fig. S3) led to decreased efficacy of LTP treatment. It was also found that increasing the amount of oxygen in the gas mixture led to decreased treatment efficacy (Fig. S2). We speculate that this is likely a result of the addition of oxygen, resulting in a decreased plume length, electron density, and ionization^25^. The pH of treated media was also tested immediately following treatment and subsequently every hour for four hours. No changes were noted in the pH of the media following LTP treatment (data not shown). Optimal settings were found to be 17–20 kV_pp_, 2 slm of He, 0.01 slm of O_2_, and ≤10 mm nozzle elevation. These optimized conditions were kept constant for all further testing. The combined data from these experiments indicate that the LTP treatments are effective against both gram-positive and gram-negative bacteria on both hard and porous surfaces.

### LTP mediates antibacterial effects by an indirect mechanism

**Figure 4:**
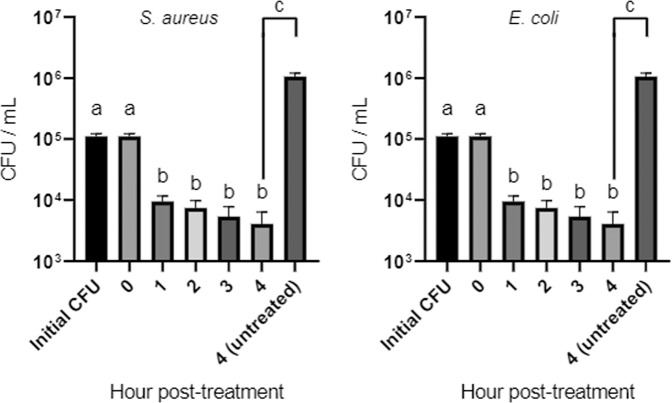
Antibacterial treatment of bacteria suspended in LB broth. Individual wells of a 24-well plate containing 1 × 10^5^ CFU/mL of *S.aureus* and *E. coli* suspended in 400 μL of LB broth were exposed to LTP for 3 minutes each. Samples were taken at 0, 1, 2, 3, and 4 hours and plated to determine CFU. Statistical significance was determined by one-way ANOVA with Tukey’s multiple-comparison test for treated samples and paired t-test for untreated sample. Significance is shown via grouping: a = significantly different from groups marked with b or c; b = significantly different from groups marked by a or c; c = significantly different from groups marked with a or b, p ≤ 0.05, N = 6.

The liquid medium creates an additional barrier to plasma due to the differential density gradient between the surrounding air, gaseous plasma, and liquid media. Therefore, we investigated the efficacy of LTP on bacteria suspended in liquid media to test the robustness of our APPJ configuration and its potential for *in vivo* use. The bacteria were suspended in LB broth and exposed to LTP at optimized conditions. Initial experiments showed that exposure to LTP did not immediately lead to a significant inhibition of bacterial growth in LB broth (data not shown). We speculated that perhaps, the effect of LTP is indirect and requires additional time to initiate the bactericidal effects. To test this hypothesis, bacteria were incubated for a period 0–4 hours following LTP treatment to determine potential time-dependent antibacterial properties of the LTP. Significant reductions in CFU of *S. aureus* (Fig. 4, left panel), and *E. coli* (Fig. 4, right panel) were observed after 1 hr of incubation following LTP treatments. Additionally, nearly a 90% reduction in CFU of both bacteria was observed by 4-hours post treatment (Fig. 4). On the other hand, both bacteria that were not exposed to LTP grew by about a log fold within 4 hr of incubation.

**Figure 5:**
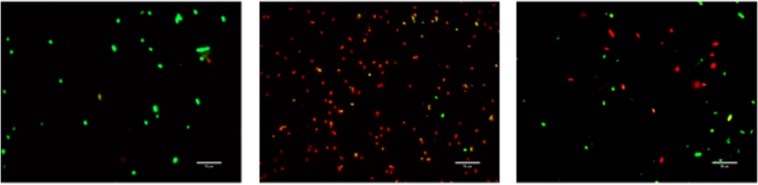
Antibacterial treatment of bacteria suspended in LB broth. Individual wells of a 24-well plate containing 2 × 10^9^ CFU/mL of *E. coli* suspended in 1 mL of LB broth were exposed to LTP for 15 minutes each. Samples were taken at 4 hours post treatment and stained using Propidium Iodide and SYTO 9. Representative images of untreated (left), heat killed (center) and plasma treated (right) samples are shown.

A fluorescent-based LIVE/DEAD *Bac*Light Bacterial Viability assay (Invitrogen, Carlsbad, CA) that differentiates live vs. dead bacteria due to their differential permeability was utilized to confirm the bactericidal effect of LTP. Samples of *E. coli* suspended in LB broth were exposed to the plasma jet, incubated for 4 hours, and then stained and imaged as per the manufacturer’s instructions. While most of the bacteria that were not exposed to LTP were green, indicating they were alive, a vast majority of the bacteria that were exposed to LTP were red, indicating they were dead. Bacteria subjected to heat treatments was used as a positive control (Fig. 5).

**Figure 6:**
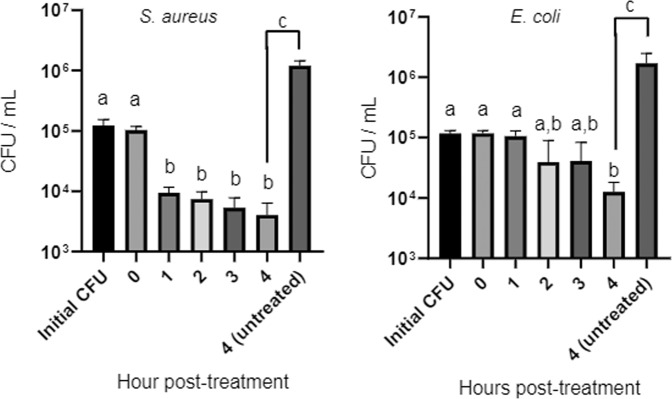
Plasma activated media (PAM) shows similar bactericidal properties to direct LTP. (**A**) Individual wells of a 24-well plate containing 400 μL of LB broth were exposed to LTP for 3 minutes each. 1 × 10^5^ CFU/mL of *S. aureus* and *E. coli* were added immediately post-treatment. Samples were incubated for 0, 1, 2, 3, and 4 hours and plated to determine CFU. Statistical significance was determined by one-way ANOVA with Tukey’s multiple-comparison test for treated samples and paired t-test for untreated sample. Significance is shown via grouping: a = significantly different from groups marked with b or c; a,b = significantly different from groups marked with c; b = significantly different from groups marked with a or c; c = significantly different from groups marked with a or b, p ≤ 0.05, N = 3.

It is important to note that there was a minimal effect of LTP on bacteria when bacteria were plated immediately after the exposure but, there was a significant effect when incubated for at least an hour after exposure. These data suggest that the bactericidal effect of LTP is indirect and a sufficient quantity of bactericidal mediators may be generated in a time-dependent manner in the media that contains bacteria. This hypothesis is supported by the previous observations that plasma – activated media (PAM), media exposed to a source of plasma and then introduced to a target sample, was effective as an aqueous disinfectant and may also have tumoricidal effects^13–16,26,27^. Therefore, we tested if PAM generated with our APPJ system would have antibacterial effects similar to that of the direct treatments. Individual wells of LB broth were directly exposed for 3 minutes each to the plasma stream. Immediately after treatment, 10^5^ CFU/mL of either *S. aureus* (Fig. 6, left panel) or *E. coli* (right panel) were added to the media, incubated for 4 hours, and plated for enumeration. It was found that treatment with PAM was able to reduce the CFU of both bacteria to levels similar to that of the direct treatment (Fig. 6). It has been shown that the conservation of bactericidal activity of PAM is dependent on storage time and conditions^22^. Therefore, we investigated the bactericidal activity of our APPJ induced PAM after specified periods of storage to determine potential changes in its physio-chemical properties. The results from these experiments confirmed a decrease in the efficacy of the PAM treatment within 2 hours of being generated (Supplementary Fig. S4). These results indicate that the bactericidal effects of the LTP treatments are mediated indirectly through the generation of labile bactericidal elements in the media, and that the PAM containing these mediators has bactericidal properties equivalent to that of direct LTP treatments.

### Reactive oxygen species are key mediators of bactericidal effects of LTP

**Figure 7:**
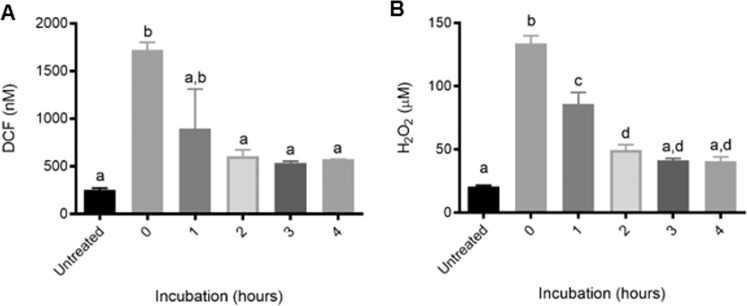
Levels of extracellular ROS/RNS during incubation period. Individual wells of a 24-well plate containing 400 μL of LB broth were exposed to LTP for 3 minutes each. Samples were incubated for 0, 1, 2, 3, and 4 hours before measuring levels of (**A**) total ROS/RNS via DCF and (**B**) H_2_O_2_. Statistical significance was determined by one-way ANOVA with Tukey’s multiple-comparison test for treated samples. Significance is shown via grouping: a = significantly from other groups marked with b, c, or d; a, b = significantly different from groups marked with c or d; b = significantly different from groups marked by a, c, or d; c = significantly different from groups marked with a, b, or d; d = significantly different from groups marked with a, b, or c; a, d = significantly different from groups marked with b or c, p ≤ 0.05, N = 3.

It has been reported that plasma can generate ROS/RNS in the presence of atmospheric oxygen and nitrogen^28,29^. Since our APPJ system incorporated gaseous oxygen into the carrier gas, and we observed the transient nature of the bactericidal mediators in PAM, we hypothesized that ROS/RNS may be generated in the media following LTP treatment and may be responsible for the killing of bacteria. Therefore, we sought to determine the generation and loss of ROS/RNS in PAM over time. Levels of total ROS/RNS and specifically H_2_O_2_ were measured by an Oxiselect *In Vitro* ROS/RNS Assay Kit (Cell Biolabs, Inc., San Diego, CA) that utilizes a dichlorodihydrofluorescin DiOxyQ probe. Total ROS/RNS, excluding H_2_O_2_, was highest at concentrations of approximately 1700 nM immediately after exposure and decreased over a period of 4-hour incubation to 600 nM (Fig. 7A). Similarly, H_2_O_2_ levels were at a maximum immediately after exposure at a concentration of 135 μM and decreased over time to 45 μM (Fig. 7B). These data show that there is a direct correlation between the levels of ROS/RNS measured over time and the efficacy of the PAM treatments that were stored for similar durations, and they suggest that ROS/RNS may be responsible for the bactericidal effects of LTP. Also of note is the fact that previous reports have shown that certain bacteria are able to produce H_2_O_2_ ^30,31^. To test for possible H_2_O_2_ contribution from the bacteria in our experiments, we measured H_2_O_2_ in both LTP treated and untreated media as well as LTP treated bacteria resuspended in fresh media. These tests conclude that there is no detectable H_2_O_2_ produced by the bacteria alone with and without exposure to LTP (Fig. S5). We also specifcally tested for RNS contribution via levels of NO_2_^−^ but were unable to detect measureable amounts in LTP treated samples (data not shown).

**Figure 8:**
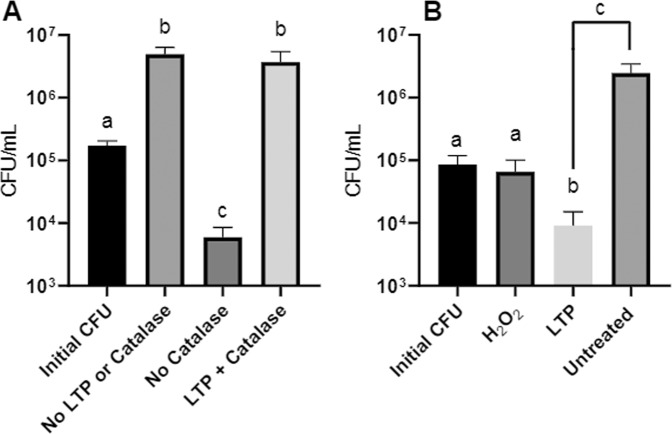
H_2_O_2_ is not sufficient for bactericidal properties of LTP. (**A**) Individual wells of a 24-well plate containing 400 μL of LB broth and 200 U of bovine liver catalase were exposed to LTP for 3 minutes each. 1 × 10^5^ CFU/mL of *E. coli* were added immediately post-treatment. Samples were incubated for 4 hours before enumerating CFU. (**B**) Wells were treated in the same manner described above with 135 μM H_2_O_2_. Samples were incubated for 4 hours before enumerating CFU. Statistical significance was determined by one-way ANOVA with Tukey’s multiple-comparison test for treated samples and two-tailed t-test for untreated sample comparison. Significance is shown via grouping: a = significantly different from groups marked with b or c; b = significantly different from groups marked with a or c; c = significantly different from groups marked with a or bp ≤ 0.05, N = 4.

To test the idea that ROS/RNS are the mediators of the bactericidal effects of LTP, bovine liver catalase, an enzyme that catalyzes the oxidation of H_2_O_2_ to inert water and oxygen, was added at 200 U/mL in LB broth prior to exposure to LTP. Addition of catalase completely inhibited the bactericidal properties of the PAM and restored *E. coli* growth back to the levels of untreated samples (Fig. 8A). These data suggest that H_2_O_2_ is essential for the bactericidal activity of LTP. We next tested if the levels of H_2_O_2_ produced following plasma treatment were sufficient for the bactericidal effects of LTP. As previously described, 135 μM of H_2_O_2_ was detected immediately following LTP treatment (Fig. 7B). Therefore, we added 135 μM of H_2_O_2_ to *E. coli* cultures and incubated for 4 hours. Surprisingly, this concentration was not sufficient to limit the bacterial growth (Fig. 8B). In fact, a minimum of 500 μM of H_2_O_2_ was required to reduce the bacterial numbers within a 4 hr window. Much higher amounts of H_2_O_2_ (15 mM) were needed to kill all bacteria present within 30 minutes (Supplementary Figs. S6 and 7). Together these data indicate that LTP treatments are able to generate multiple reactive species including H_2_O_2_. Additionally, they show that H_2_O_2_, is essential but not sufficient to limit bacterial growth, and that other more transient reactive species likely play a significant role.

### Bacteria do not develop resistance to LTP treatments

**Figure 9:**
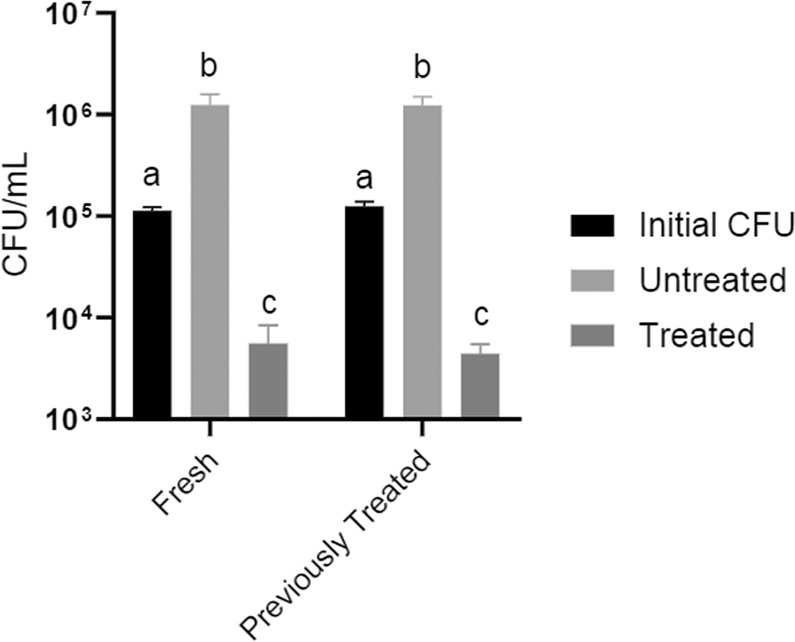
Effectiveness of LTP treatment on fresh and previously treated bacteria. Individual wells of a 24-well plate containing 1 × 10^5^ CFU/mL of fresh and previously treated *E. coli* from prior experiments suspended in 400 μL of LB broth were re-exposed to LTP for 3 minutes each. Re-exposure was repeated 4 times. Samples were incubated for 4 hours before enumerating CFU. Statistical significance was determined by two-way ANOVA with Tukey’s multiple-comparison test. Significance is shown via grouping: a = significantly different from groups marked with b or c; b = significantly different from groups marked with a or c; c = significantly different from groups marked with a or b, p ≤ 0.05, N = 4.

Finally, to test if bacteria develop resistance to LTP treatment, surviving bacteria following LTP treatments were re-exposed to LTP. Samples of treated *E. coli* from previous direct exposure experiments were inoculated into 5 mL of fresh LB and regrown to log phase. These bacteria were then re-exposed to direct LTP treatment as previously described. Figure 9 shows that there was no significant difference in the susceptibility to LTP between the previously unexposed and bacteria that had been re-exposed 4 times. These data indicate that LTP treatment is equally effective with bacteria that was previously exposed. Therefore, this supports the idea that LTP treatments have the potential to serve as a superior alternative to antibiotic treatments because of the decreased potential for the development of bacterial resistance.

## Discussion

The appeal of APPJs and other low-temperature plasma sources for future therapeutic applications are multi-fold. These sources are non-thermal and therefore, can be applied to living tissue without thermal damage. They produce complex plasma-induced reactive chemistry, such as reactive oxygen and nitrogen species (RONS), that result in anti-bacterial effects, as shown in this work and others. This “dry” chemistry does not rely on liquid chemicals for anti-bacterial treatment. Critically, several reports have demonstrated the efficacy of plasma sources against antibiotic-resistant bacteria^32^ as well as against biofilms^32^. Additionally, there are no indications of the evolution of bacterial resistance to the plasma treatment^33–36^.

By using the APPJ configuration, we were able to demonstrate that LTP treatments can generate ROS/RNS in a localized environment, leading to the inhibition of growth of both gram-positive and gram-negative bacteria on solid surfaces, porous surfaces with distributed bacteria, as well as in liquid media. Neutralization of H_2_O_2_ abrogated the antibacterial effects of LTP, indicating that H_2_O_2_ is necessary for the bactericidal activity of LTP. Interestingly, addition of exogenous H_2_O_2_ at equal concentration was not sufficient to induce effects similar to LTP treatments, indicating that other more transient reactive species play an important role. On the other hand, a similar effect was observed in the PAM generated by the APPJ configuration suggesting that both direct APPJ LTP exposure or PAM have potential for a variety of clinical applications.

It is well accepted that multiple types of ROS/RNS are generated from LTP treatments^26,29^. However, the location of formation of the reactive species, and their ability to diffuse into porous surfaces and the liquid media from the gaseous plasma phase for APPJ configurations has not been as clearly established. Electron paramagnetic resonance spectroscopy and proton nuclear magnetic resonance analysis have revealed that more stable species such as hydrogen peroxide are largely formed within the tube of the plasma jet, and that more reactive and transient species such as hydroxyl and superoxide tend to form in the plasma stream outside of the tube and between the nozzle of the jet and the target sample^29^. Additionally, the amounts of different types of species generated as a function of various system parameters is also under investigation. For example, it has been reported that the composition of the carrier gas, specifically the inclusion of oxygen is important in the generation of species such as O_3_, O_2_, and O^27^. Therefore, the location of formation of free radicals and the types of radicals that are able to be produced are key regulators of the interactions of the LTP with the treatment target. Based on these reports, we hypothesized that our APPJ configuration and the use of oxygen in our carrier gas mixture would generate significant amounts of H_2_O_2_ in addition to a variety of other free radical species. Indeed, our results indicated that ROS, including H_2_O_2_ are generated in treated samples (Fig. 7). Further experiments using more time-sensitive assays could give a better understanding of the various types of ROS/RNS that are present throughout and after treatment of the sample with LTP.

It was determined that H_2_O_2_ was necessary but not sufficient to reach the level of bactericidal activity produced by the LTP treatments (Figs. 7 and 8). There are several potential explanations for these results. As previously discussed, LTP treatments are thought to produce both short- and long-lived reactive species in both gaseous and aqueous forms^37^. We measured only stable ROS (Fig. 7), but the effects of the difficult to quantify, short-lived products were not directly investigated. Additionally, measurement of NO_2_^−^ with a Griess assay showed that amounts were below the limit of detection. Therefore, it is possible that short-lived ROS/RNS generated by LTP treatments play a significant role in combination with H_2_O_2_ to generate the bactericidal properties observed.

An alternative explanation could be interaction of UV irradiation with H_2_O_2_. It has been shown that small amounts of UV radiation are emitted by LTP devices, but it is thought that the dose is not sufficient to cause damage to the targeted treatment sample and that the majority of the VUV photons (<180 nm) are absorbed into the air^29,38^. However, H_2_O_2_ can absorb large amounts of UV energy at short wavelengths, resulting in its dissociation into highly reactive, antibacterial hydroxyl radicals^28,29,39–41^. Therefore, it is possible that at the time of LTP treatment, far more potent, hydroxyl radicals are generated. The formation of these radical species could explain the superior efficacy of LTP treatments in comparison with direct H_2_O_2_ treatment. On the other hand, persistence of antibacterial activity of PAM for at least two hours suggest the contribution of such highly-reactive species may be limited. Therefore, further research is required to examine any potential synergistic effects between ROS and UV energy generated within the LTP treatments.

Since we observed a minimum requirement of a 2-hour incubation for effective bacterial killing following LTP treatment, we proposed that the bactericidal elements were accumulating in the media over time (Fig. 4). However, this hypothesis was refuted by evidence demonstrating the labile properties of the bactericidal effects of PAM (Supplementary Fig. S4). Therefore, we speculate that the 2-hour incubation is necessary for cellular damage to occur. Previous reports have shown that very high concentrations of ROS (>1 mM), require minimal exposure times to kill bacteria via peroxidation of lipids, DNA, and proteins^41^. In our case, ROS levels are highest immediately after LTP treatment but are still approximately ten times lower than concentrations reported to immediately exert inhibition of bacterial growth. This disparity could explain the necessity of the incubation period for gradual and progressive damaging effects of ROS. It is also noteworthy that levels of H_2_O_2_ produced by bacteria in previous reports are significantly lower (by approximately 1000-fold)^30^ than the levels that we observe following LTP treatment and therefore unlikely to contribute to cell damage.

Our results also consistently showed that LTP treatments have slightly increased efficacy against *S. aureus* in comparison to *E. coli*. There have been mixed reports as to whether gram-positive or gram-negative bacteria are more strongly affected by various types of plasma treatments. Mai-Prochnow *et al*. demonstrated a correlation between LTP inactivation of bacteria with cell wall thickness, indicating that gram-negative species are more susceptible than gram-positive species. The plasma source used for these experiments was a kINPen med (Neoplas tools GmbH Greifswald, Germany)^8^ coupled with an argon feed gas, and a pulse generator set to a frequency of 1.82 MHz^18^. Alternatively, Tipa *et al*. showed that LTP treatments using compressed air in conjunction with a 13.56 -MHz micro-jet appeared to be more effective against gram-positive bacteria in comparison with gram-negative bacteria. These findings were attributed to the protective qualities of the outer membrane associated with only gram-negative species^19^. It is plausible that the disparities in these results are due to differences in discharge configurations. As shown in Supplementary Figs. S1–3, variations in components of the system such as carrier gas, electrode configuration, voltage magnitude, and frequency of current can result in altered bactericidal activity of the LTP treatment. Therefore, we hypothesize that these factors impact the composition of reactive species being generated by the LTP. Additionally, for our plasma source, we implemented a carrier gas mixture of oxygen and helium which would be more comparable to the compressed air experiments by Tipa *et al*. Therefore, it is perhaps not surprising that we noted differential susceptibility between gram-positive and gram-negative bacteria similar to that reported by Tipa *et al*.

Since the bactericidal effect of LTP was mediated through ROS, another potential reason for the observed difference in response of *S. aureus* in comparison with *E. coli* could be due to dissimilar levels of antioxidant mechanisms. There have been multiple reports indicating variation in antioxidant machinery in different bacterial strains^42–45^. Differential production of enzymes such as catalase, superoxide dismutase, and glutathione peroxidase by the bacteria could explain differences in treatment efficacy against various bacterial strains. This is in fact supported by the results demonstrating that the addition of catalase abrogates the bactericidal effects of the LTP treatment (Fig. 8A). However, further research will be required to more clearly elucidate the relationship between antioxidant mechanisms and differential susceptibility to LTP treatment in bacteria.

These studies have many potential applications within the field of plasma medicine and will be useful in the development of innovative, alternative strategies for antiseptics. Direct treatment using the methods discussed here could be applied for surface level infections and in porous materials, while PAM treatments could be used to target tissue specific, localized infections within the host. Finally, further investigation of the mechanisms of non-H_2_O_2_ dependent bactericidal properties suggested in this paper may prove useful for identifying previously undiscovered targets for new antibiotic therapies.

## Materials and Methods

### Bacterial strains

*Escherichia coli*: *E. coli* K12 cells were grown in Luria-Bertani (LB) Broth, Miller (BD Difco Laboratories, Detroit, MI) to stationary phase. Cells were harvested and resuspended at 10^5^ CFU/mL in LB Broth. After treatment, cells were incubated at 37 °C for specified times and then plated for enumeration of CFU on LB agar. *Staphylococcus aureus*: *S. aureus* USA300 JE2 cells were grown in LB Broth to stationary phase. Cells were harvested and resuspended at 10^5^ CFU/mL in LB Broth. After treatment, cells were incubated at 37 °C for specified times and then plated for enumeration of CFU on LB agar.

### APPJ device

A diagram of the plasma jet is shown in Fig. 1. The APPJ was constructed from a quartz capillary tube of 4 mm outer diameter and 2.4 mm inner diameter. Two outer ring copper electrodes were placed 10 mm apart on the tube and excited by a high-voltage AC power supply. A 5 kHz sine wave at 17 kV_pp_–20 kV_pp_ was applied to the upstream electrode, and the downstream electrode was grounded. Helium gas from 1–2 standard liters per minute (slm) and oxygen gas from 0.01–0.08 slm were used for operation, metered through mass flow controllers (MKS Instruments, Inc.). The APPJ was centered above the treatment target at nozzle elevations between 10–30 mm. The plasma plume was visible during operation.

The high-voltage amplifier is a Trek Inc. Model 10/40 with sinusoidal voltage capability up to ±10 kV amplitude at up to 20 kHz. Voltage measurements from the amplifier and across the monitor capacitor were made on a Tektronix oscilloscope with 100 MHz bandwidth (DPO2012B). The power delivered to the plasma was evaluated using the Lissajous method, measuring the voltage across a monitor capacitor (*C* = 10 nF) placed, in series, between the downstream electrode and the electrical ground of the high-voltage amplifier.

### Solid agar plate assay

*S. aureus* and *E. coli* were plated at 2 × 10^8^ and 1.5 × 10^8^ CFU/mL, respectively, on LB agar plates and left to rest for 20 minutes. Sinusoidal voltages of 20 kV_pp_ at a frequency of 5 kHz were applied to the APPJ. Gas flow rates of 2 slm for helium and 0.01 slm for oxygen were used. Individual plates were exposed to the plasma stream for 0–120 seconds. Plates were incubated overnight at 37 °C and then imaged. Zones of inhibition were quantified using ImageJ, which calculates the area of selection in square pixels and in the calibrated unit of choice. Additionally, each image was spatially calibrated using the ratio of the area of inhibition to the area of the petri dish plate.

### Soft agar plate assay

*S. aureus* and *E. coli* were added at 5 × 10^7^ CFU/mL to a mixture of one-part LB agar and three parts LB broth. The mixture was plated on LB agar plates and left to rest for 20 minutes. Sinusoidal voltages of 20 kV_pp_ at a frequency of 5 kHz were applied to the APPJ. Gas flow rates of 2 slm for helium and 0.01 slm for oxygen were used. Individual plates were exposed to the plasma stream for 0–120 seconds. Plates were incubated overnight at 37 °C and then imaged. Zones of inhibition were quantified using ImageJ, which calculates the area of selection in square pixels and in the calibrated unit of choice. Additionally, each image was spatially calibrated using the ratio of the area of inhibition to the area of the petri dish plate.

### Liquid culture assay

*S. aureus* and *E. coli* were added at 1 × 10^5^ CFU/mL to 400 μL of LB broth in 24-well plates. Sinusoidal voltages of 17 kV_pp_ at a frequency of 5 kHz were applied to the APPJ. Gas flow rates of 2 slm for helium and 0.01 slm for oxygen were used. Individual wells were exposed to the plasma stream for 180 seconds. The plate was incubated for 0–4 hours. Samples were plated on LB agar for enumeration of CFU.

### Secondary treatment assay

*E. coli* was added at 1 × 10^5^ CFU/mL to 400 μL of LB broth in 24-well plates. Sinusoidal voltages of 17 kV_pp_ at a frequency of 5 kHz were applied to the APPJ. Gas flow rates of 2 slm for helium and 0.01 slm for oxygen were used. Individual wells were exposed to the plasma stream for 180 seconds. The plate was incubated for 4 hours. 100 μL of treated samples were inoculated into 5 mL of fresh LB and regrown to log phase. Re-cultured samples in addition to fresh bacteria were added at 1 × 10^5^ CFU/mL to 400 μL of LB broth in 24-well plates. Individual wells were again exposed to the plasma stream for 180 seconds. The plate was incubated for 4 hours. Samples were plated on LB agar for enumeration of CFU.

### Measurement of ROS/RNS/hydrogen peroxide

ROS/RNS analysis was performed using an OxiSelect *In Vitro* ROS/RNS Assay (Cell Biolabs, San Diego, California, USA) as per manufacturer’s instructions. The mean fluorescent intensity at 480 nm (excitation) and 530 nm (emission) were measured using a fluorescence plate reader (SpectraMax iD3, Molecular Devices, San Jose, CA, USA).

A secondary method of hydrogen peroxide analysis was performed using an Amplex Red Hydrogen Peroxide/Peroxidase Assay (ThermoFisher Scientific, Waltham, Massachusetts, USA) as per manufacturer’s instructions. The mean fluorescent intensity at 530 nm (excitation) and 590 (emission) were measured using a fluorescence plate reader (SpectraMax iD3, Molecular Devices, San Jose, CA, USA).

### Statistics

Statistical analyses were performed with GraphPad Prism version 8.01 for Windows (GraphPad Software, La Jolla, California, USA). Data are shown as mean ± standard deviation unless otherwise stated. One-way or two-way analysis of variance (ANOVA) coupled with the Tukey post hoc tests and one-sided t-tests were used to determine effects for measured variables and locators of significance difference. Statistical significance was set at p < 0.05.

## Supplementary information

Supplementary Information.
