# Supplementary material for: Antibacterial effects of low-temperature plasma generated by atmospheric-pressure plasma jet are mediated by reactive oxygen species
Source: Sci Rep. 2020 Feb 20;10:3066. doi: 10.1038/s41598-020-59652-6 (PMC7033188; doi:10.1038/s41598-020-59652-6)
Supplement: Supplementary file 1 — Supplementary Information. [file 41598_2020_59652_MOESM1_ESM.docx]

**Supplementary Figures:**

**
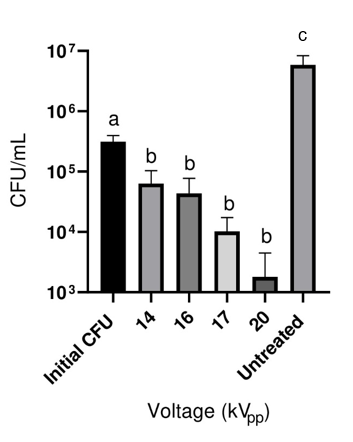
**

**Figure S1: Optimization of voltage of APPJ.** Individual wells of a 24-well plate containing 1x10^5^ CFU of *E. coli* suspended in 400 µL of LB broth were exposed to LTP for 3 minutes at varying voltages. Samples were incubated for 4 hours and CFU were enumerated. Statistical significance between treated samples was determined by one-way ANOVA with Tukey’s multiple-comparison test. Significance is shown via grouping: a = significantly different from groups marked with b or c; b = significantly different from groups marked with a or c; c = significantly different from groups marked with a or b, p≤0.05, N=6.


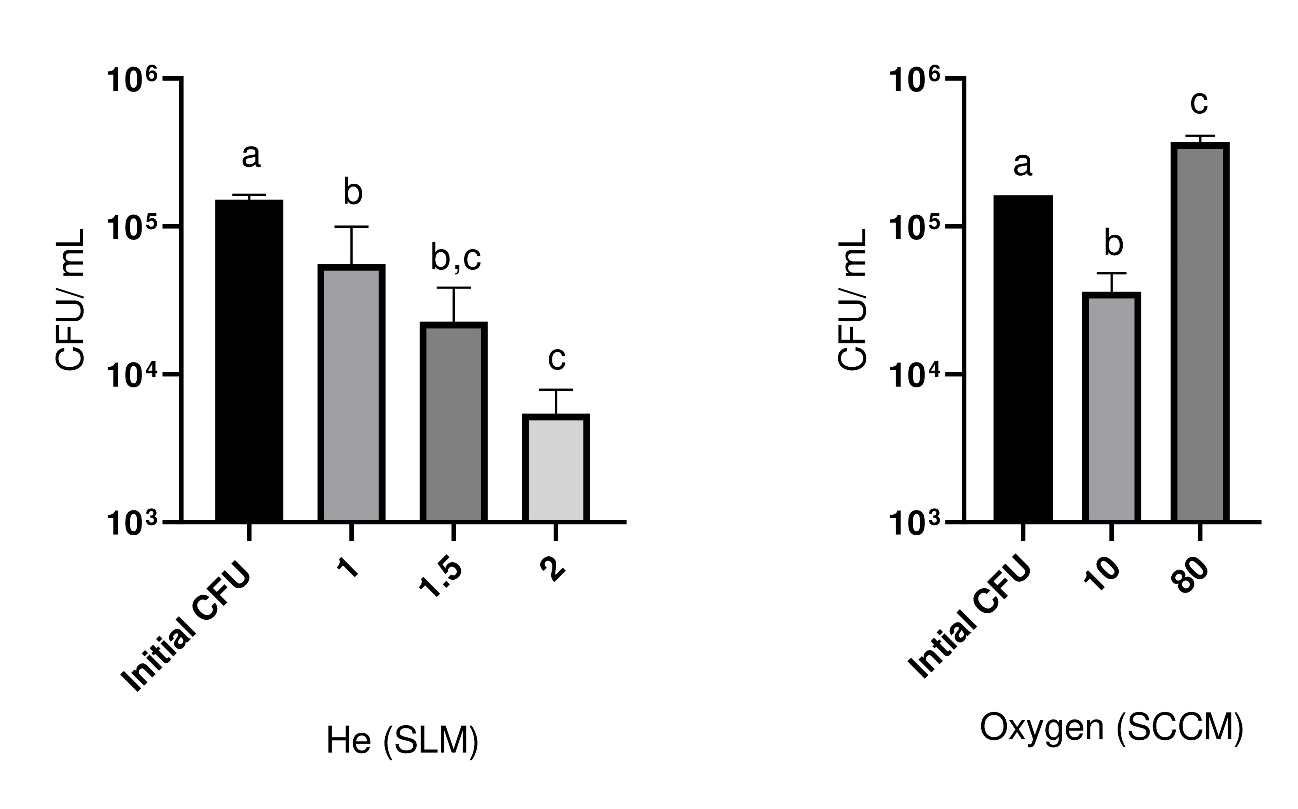


**A**

**B**

A

**Figure S2: Optimization of gas flow rate and mixture ratios.** Individual wells of a 24-well plate containing 1x10^5^ CFU of *E. coli* suspended in 400 µL of LB broth were exposed to LTP for 3 minutes. **A)** Flow rate of helium was varied while holding oxygen constant at 0.01 slm. Samples were incubated for 4 hours and CFU were enumerated. **B)** Flow rate of oxygen was varied while holding helium constant at 1 slm. Samples were incubated for 4 hours and CFU were enumerated. The reduced effect associated with the 0.08 slm oxygen mixture compared to 0.01 slm is likely due to a shortening of the plasma jet and reduced interaction with the liquid at the same distance from the jet nozzle to the plate. Statistical significance was determined by one-way ANOVA with Tukey’s multiple-comparison test. Significance is shown via grouping: a = significantly different from groups marked with b or c; b = significantly different from groups marked with a or c; c = significantly different from groups marked with a or b; b,c = significantly different from groups marked a, p≤0.05, N=3.


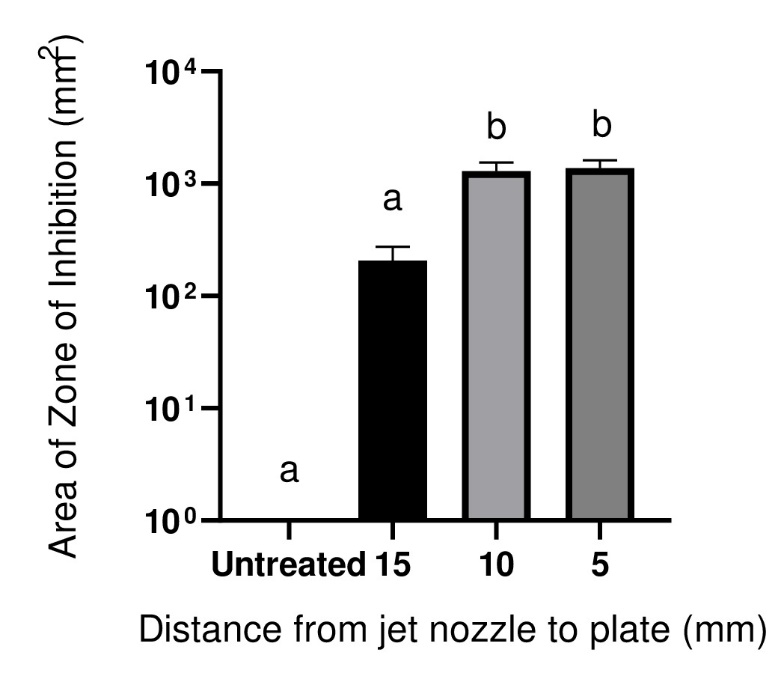
**Figure S3: Optimization of distance between jet nozzle and sample.** LB agar plate with 5x10^7^ CFU of *E. coli* were exposed to LTP for 90 seconds. Plates were incubated overnight and quantification of area of zone of inhibitioon was done using ImageJ. Statistical significance was determined by one-way ANOVA with Tukey’s multiple-comparison test. Significance is shown via grouping: a = significantly different from groups marked with b; b = significantly different from groups marked with a, p≤0.05, N=3.


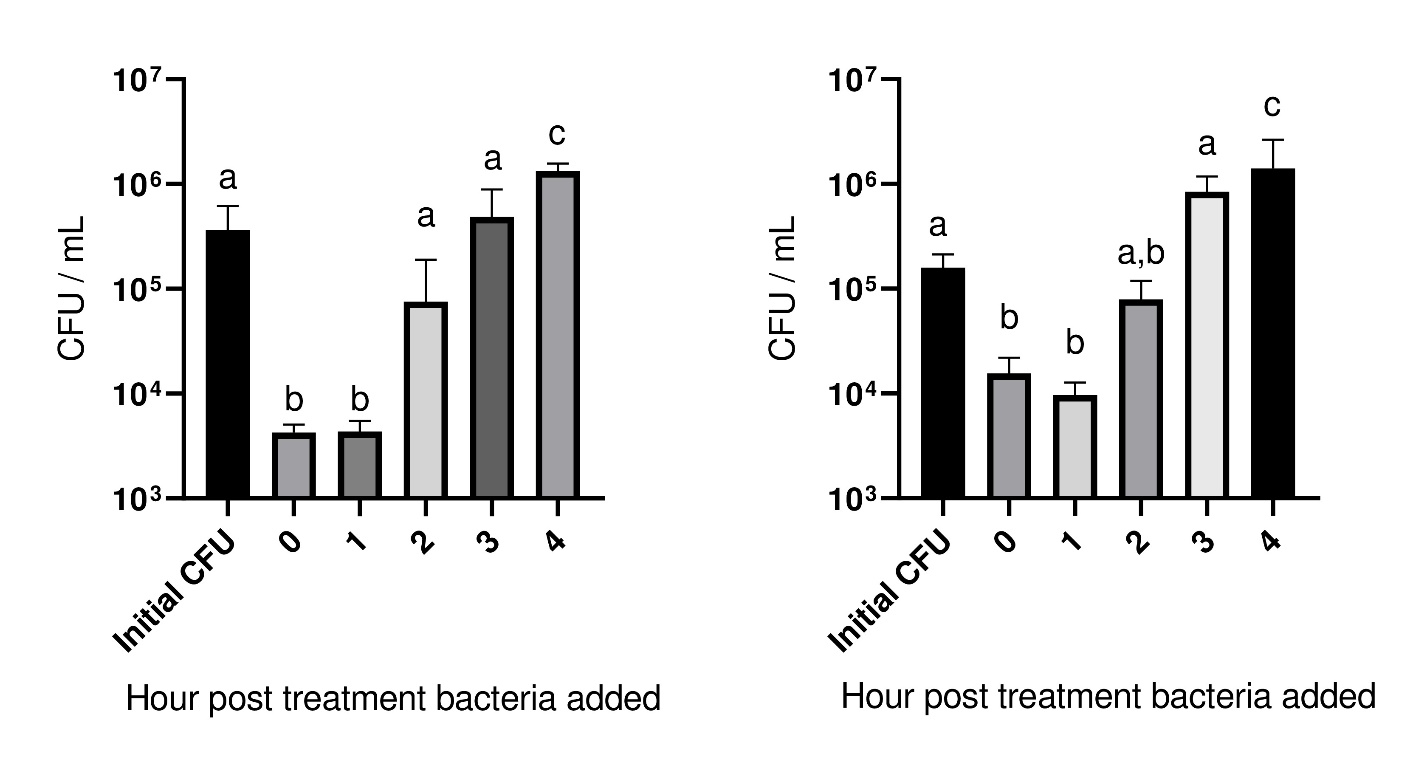


*S. aureus*

*E. coli*

**Figure S4: Labile properties of PAM.** Individual wells of a 24-well plate containing 400 µL of LB broth were exposed to LTP for 3 minutes. At specified time points following treatment, 1x10^5^ CFU of *S. aureus* (left) or *E. coli* (right) were added. Samples were then incubated for an additional 4 hours after the addition of bacteria, and CFU were enumerated. Statistical significance was determined by one-way ANOVA with Tukey’s multiple-comparison test. Significance is shown via grouping: a = significantly different from groups marked with b or c; b = significantly different from groups marked with a or c; a,b = significantly different from groups marked with c; c = significantly different from groups marked with a or b, p≤0.05, N=3.


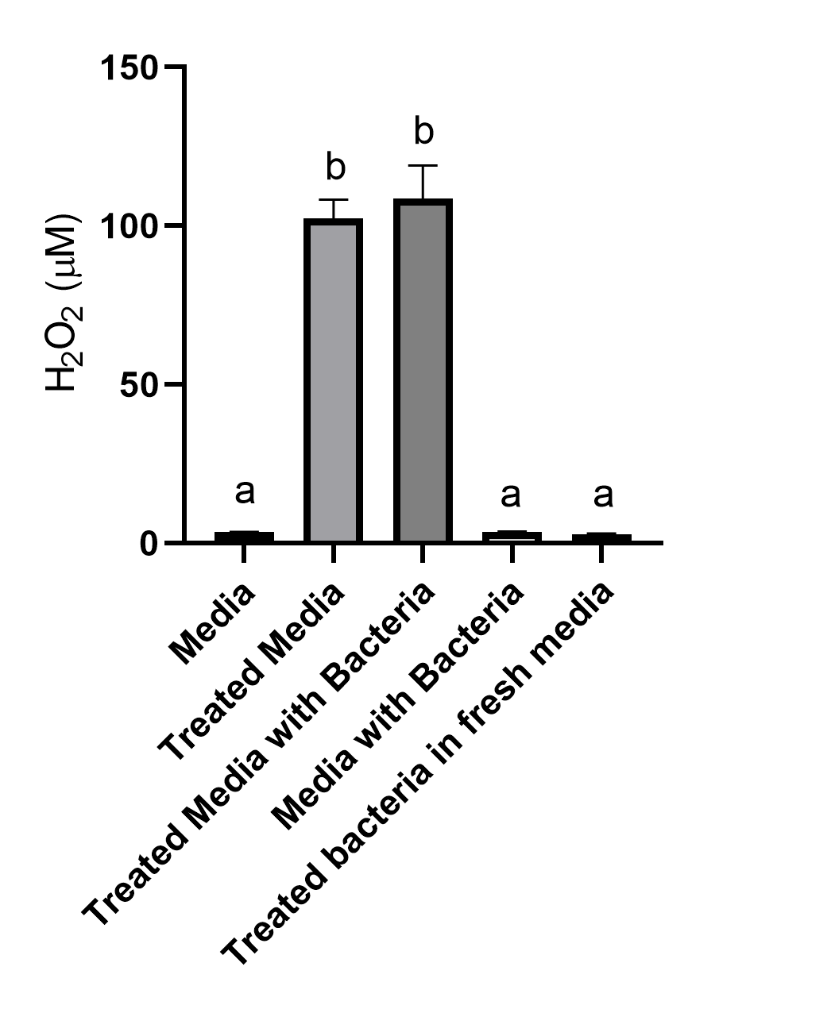


**Figure S5: Bacteria does not contribute to H_2_O_2_ levels.** Individual wells of a 24-well plate containing 400 µL of LB broth or 400 µL of LB broth with 1x10^5^ CFU of *E. coli* were exposed to LTP for 3 minutes. Immediately following treatment, some of the bacteria that had been treated was removed from the treated media and re-suspended in fresh media. Levels of H_2_O_2_ were then measured for all samples. Statistical significance was determined by one-way ANOVA with Tukey’s multiple-comparison test. Significance is shown via grouping: a = significantly different from groups marked with b; b = significantly different from groups marked with a, p≤0.05, N=3.


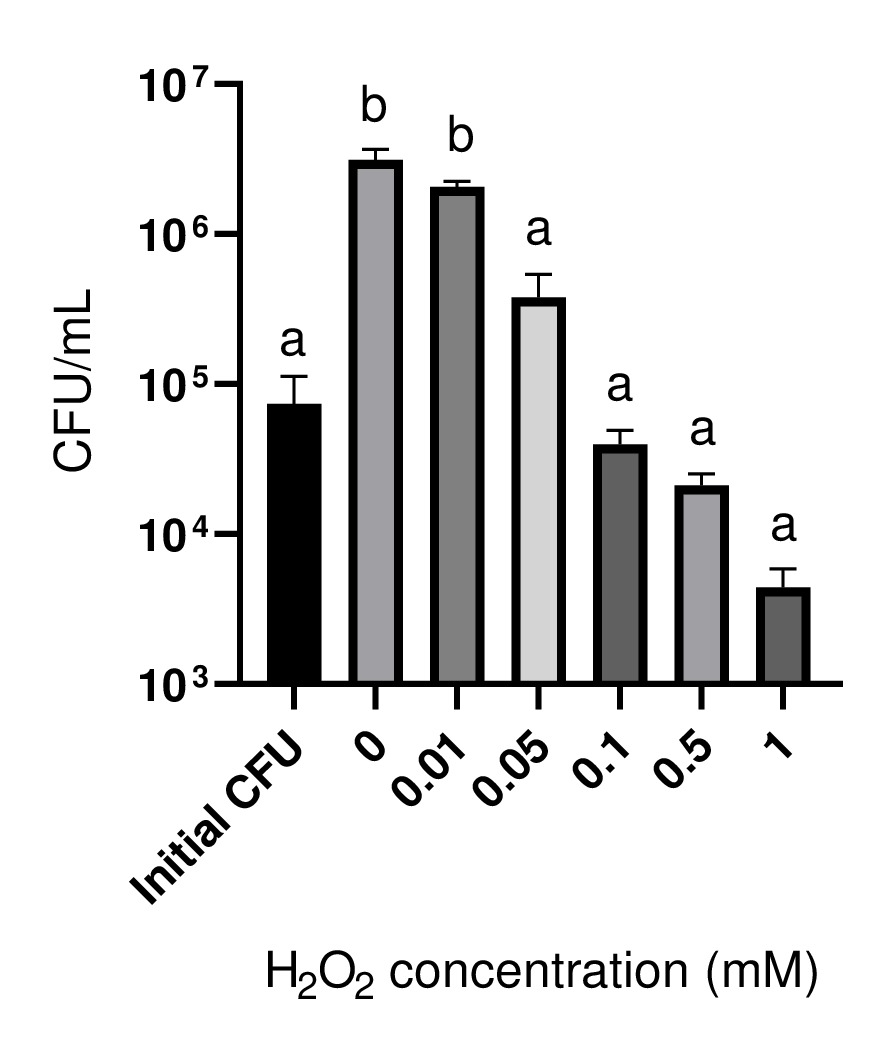


**Figure S6: Dose dependent response of *E. coli* to exogenous H_2_O_2_.** Individual wells of a 24-well plate containing 1x10^5^ CFU of *E. coli* suspended in 400 µL of LB broth were treated with varying concentrations of H_2_O_2_. Samples were incubated for 4 hours and CFU were enumerated. Statistical significance was determined by one-way ANOVA with Tukey’s multiple-comparison test. Significance is shown via grouping: a = significantly different from groups marked with b; b = significantly different from groups marked with a, p≤0.05, N=3.


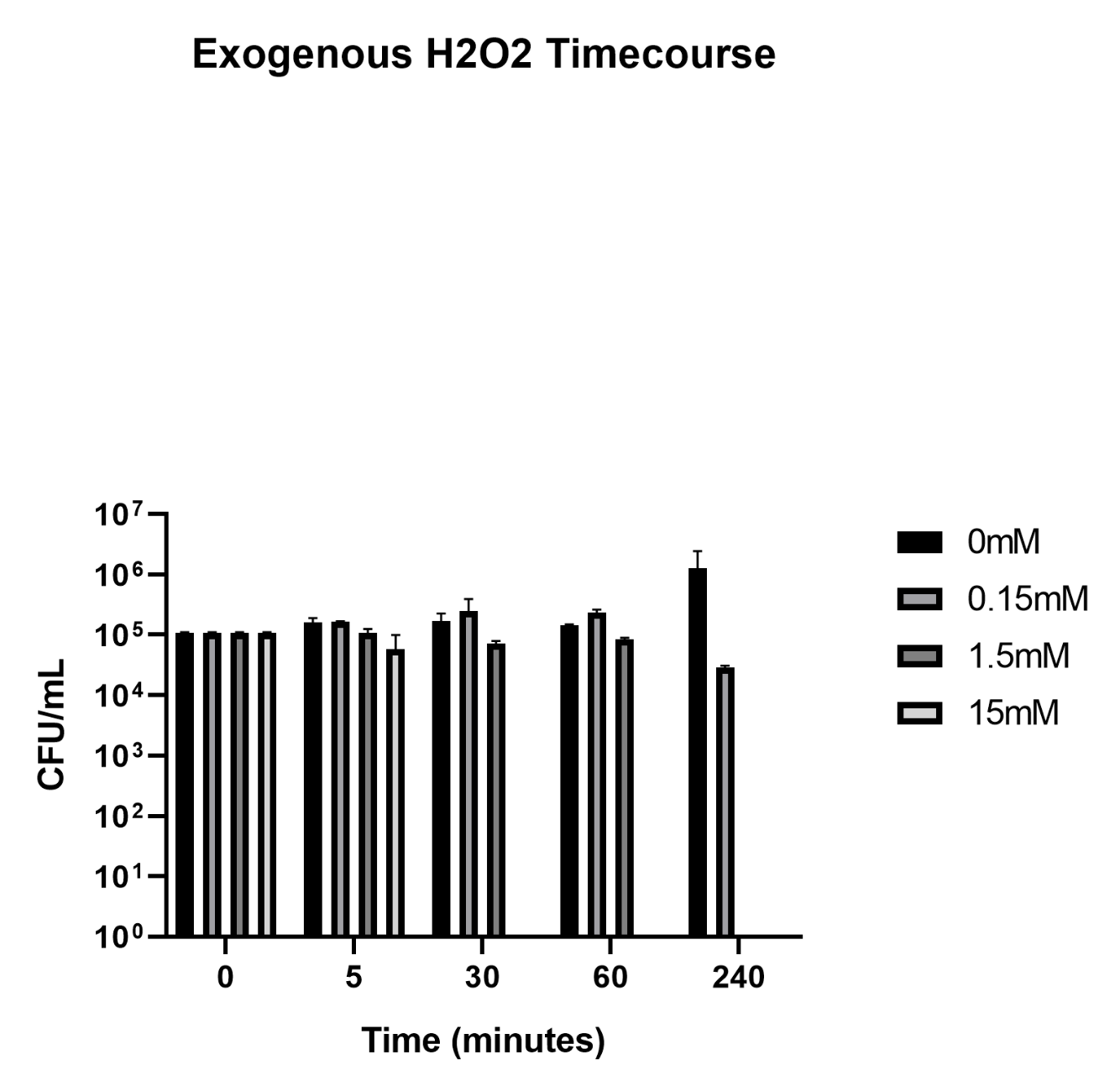


**Figure S7: Time dependent response of *E. coli* to different doses of exogenous H_2_O_2_.** Individual wells of a 24-well plate containing 1x10^5^ CFU of *E. coli* suspended in 400 µL of LB broth were treated with varying concentrations of H_2_O_2_. Samples were incubated for the indicated times and CFU were enumerated, N=3.
